# Medical News and Miscellany

**Published:** 1893-05

**Authors:** 


### Medical News and Miscellany.

---

**Dr. W. G. Jameson**, Chief Surgeon of the I. & G. N. Railroad at Palestine, is at the New Orleans Polyclinic.

---

**Dr. A. W. Fly**, President Galveston County Medical Society, is a candidate for mayor of Galveston and will doubtless be elected.

---

**Prof. Seth M. Morris**, M. D., Professor of Chemistry, Texas Medical College, has been re-elected by the Board of Regents at the recent annual meeting held in Galveston.

---

**Dr. H. C. Grace.** The JOURNAL is pained to learn of the death, at Oakwood, Texas, recently, of Dr. Harry C. Grace, a talented physician and an old subscriber to the JOURNAL.

---

**Prof. J. E. Thompson**, M. D., Professor Surgery in the Texas Medical College, (Med. Dep. Univ. Texas,) will leave for Europe very shortly and will be absent three months, returning by the first of September.

---

The Belgian Academy of Medicine has offered a prize of 4000 francs ($800) for the best essay upon the pathology and treatment of epilepsy. Papers in competition must be presented before February 1894.—*Ex.*

---

**Dr. J. H. Sears**, of Waco, the newly elected President of the Texas State Medical Association, was one of the organizers of the Association (1869). His election gives very general satisfaction; it is a merited compliment.

---

**Another Four-Year School.**—The Women's Medical College of Pennsylvania has advanced the requirements for graduation to four years. All new matriculants must now take four annual courses of lectures. Thus of Philadelphia's five medical schools, two have recognized the demands for advanced medical training.—*Ex.*

---

**Honors to American Physicians Abroad.**— Professors Horatio C. Wood, Edward T. Reichert, Hobart A. Hare, of Philadelphia, and Allen J. Smith, of Galveston, have been made Corresponding Fellows of the Sociedad Española de Higiene, of Madrid, in recognition of their valuable contributions to medical science.

---

**Dr. J. B. S. Holmes,** of Rome, Ga., whose infirmary was burned in January, has leased some residences and had them fitted up for temporary use as an infirmary and is now, May 10, ready for the reception of patients. His infirmary is being rapidly re-built. Dr. Holmes' fame as a surgeon-gynecologist is too well known to require mention in this connection.

---

**For Sale.**—Dr. W. B. Anderson, whose card appeared in the JOURNAL last winter, now, since his expected trip is close at hand, offers his property for less than cost. This is certainly an unusual opportunity, for the Doctor can put a physician into an annual $2000 practice at once without opposition. Address him at CONTENT, RUNNELS CO., TEXAS.

---

**Triplets.**—Dr. R. H. Eanes, of Taylor, Texas, reports to the JOURNAL a case of triple birth in his practice. Mrs. W., second labor, first a boy, gave birth to three boy children on the 2d of May inst. The birth was premature by six weeks, and the children died. "With the first two, the head presented; third, breech presentation. No difficulty or delay in delivery. Weight, 2½, 3 and 3½ pounds respectively."

---

Our attention has been called to an article "The Nostrum Cancer" in the March number of *Merck's Bulletin*—the Hessian trade journal of Merck's drug house, Hesse Darmstadt, Germany. Is the German a better chemist than the American? Is not the reverse true? Are the German products advertised in this German trade journal any more ethical than those made by the American chemist and advertised in medical journals? Glance at the articles advertised by German chemists in this trade journal; what do you learn about their formulæ? and how to make them? Examine carefully and draw your own conclusions. Are the medical journals of America more venal and ignorant than the German medical journals? Is the German doctor a better physician than the American? Is it not time for the medical journals of this country to assert their Americanism, and sit down on this German impudence?

---

Surgical Instruments.—A recent number of Meyer Bros. & Co.'s St. Louis *Drug Magazine* states that the Fort Worth Pharmacy Company, of Fort Worth, carries the largest stock of Surgical Instruments and mechanical curative devices in the Southwest. We are personally acquainted with these people and have a correct knowledge of their stock, fully endorse the above, and will further say, their prices we know to be as low as any of the Eastern houses, and that they are now giving good satisfaction to five hundred Texas doctors whom they number as their patrons. A year ago we asked the profession to aid us in building up this house—a home institution—where orders could be promptly filled and delivered. We have now to say that satisfactory results are being obtained. The Fort Worth Pharmacy Company are also agents for three manufactories of Electric Batteries and two manufactories of Physicians' Chairs. Write to them for what you may want.

---

**Beauties of the English.**—Fastidious, was Doctor DeLisle; he affected the "heavy-swell" stysle; when a lady he'd meet in crossing the street, he'd dexterously doff his new tisle.

A sharper, was Doctor Carlisle; tho' his face was all innocent of guisle. He'd back "a small pair," with a nonchalant air, and rake in the whole of the pisle.

Lazy was young Doctor Wright. He hated to get up at wnight; but when he did go he made a great show, and knocked the case higher than a wkight.

Busy was old Doctor Pugh; he had more'n he sometimes could dugh. But he went day and night, for all 't was in sight, and of funerals,—well, he had not a fugh.

Then there was a new Doctor named Talliafero.* He hailed from somewhere up about Bolliafero. He drove an odd pair,—a horse and a mare, and his "cub" was a boy they called Oliaferro!

C. U. LATER, M. D.

---

*Pronounced "Tolliver."—ED.

